# A-Systematic Review of the Level of Mental Health Literacy Among University Students Regarding Seeking Support From Counsellors in the UK

**DOI:** 10.1192/bjo.2022.244

**Published:** 2022-06-20

**Authors:** Areej Serebel

**Affiliations:** University of East London, London, United Kingdom

## Abstract

**Aims:**

Introduction: Mental health literacy (MHL) is defined as “one's knowledge and beliefs about mental disorders” (Nishida-Hikiji et al., 2021). Additionally, it includes the capability to recognise specific disorders, distinguish risk factors and causes, recognise self-treatments and available professional help, and it is an attitude that encourages recognition and appropriate help-seeking (Jorm et al., 1997). Background: In the UK mental health problems are one of the main public health issues as it affects one in four individuals. It specifically has a high prevalence among university students which is between the ages of 16–24 years (Kessler et al., 2005)

**Methods:**

The study design used for this study was a systematic literature review in which data were collected from PubMed, EBSCO and ScienceDirect using specific keywords in the advanced search. The amount of papers found were 953 after abstract screening for keywords only 34 papers were left and then final abstract and full-text article screening for the inclusion and exclusion criteria was done leaving it with only 8 papers. A quality assessment was done for the eight papers using CASP tool for RCT papers and EPHH tool for cross-sectional papers.

**Results:**

The findings showed that there are different levels of MHL between university students depending on their gender, education year, faculty and ethnicity. It was also found that the professional help seeking behaviour is not significantly different between genders but there was a difference regarding those identifying themselves as bisexuals despite them having high MHL scores.

**Conclusion:**

In conclusion the MHL of university students in the UK was found to be lower than those in Australia thus stating that more attention should be drawn to the issue and the need for easily accessible counselling services should be provided and promoted by universities. So that students can know about their existence and make good use of it whenever needed.

